# Viral-induced neuroinflammation: Different mechanisms converging to similar exacerbated glial responses

**DOI:** 10.3389/fnins.2023.1108212

**Published:** 2023-03-02

**Authors:** Brenda Rocamonde, Uzma Hasan, Cyrille Mathieu, Hélène Dutartre

**Affiliations:** ^1^Centre International de Recherche en Infectiologie, Équipe d’Oncogenèse Rétrovirale, INSERM U1111 - Université Claude Bernard Lyon 1, CNRS, UMR 5308, École Normale Supérieure de Lyon, Université Lyon, Lyon, France; ^2^Equipe Labellisée par la Fondation pour la Recherche Médicale, Labex Ecofect, Lyon, France; ^3^Centre International de Recherche en Infectiologie, Team Enveloped Viruses, Vectors and Immunotherapy INSERM U1111 - Université Claude Bernard Lyon 1, CNRS, UMR 5308, École Normale Supérieure de Lyon, Université Lyon, Lyon, France; ^4^The Lyon Immunotherapy for Cancer Laboratory (LICL), Centre de Recherche en Cancérologie de Lyon (CRCL, UMR INSERM 1052 – CNRS 5286) Centre Léon Bérard, Lyon, France; ^5^Centre International de Recherche en Infectiologie Équipe Neuro-Invasion, Tropism and Viral Encephalitis, INSERM U1111 - Université Claude Bernard Lyon 1, CNRS, UMR 5308, École Normale Supérieure de Lyon, Université Lyon, Lyon, France

**Keywords:** neuro-infection, HTLV-1, measles virus, Nipah virus (NiV), HAM/TSP pathogenesis, multiple sclerosis, encephalitis

## Abstract

There is increasing evidence that viral infections are the source/origin of various types of encephalitis, encephalomyelitis, and other neurological and cognitive disorders. While the involvement of certain viruses, such as the Nipah virus and measles virus, is known, the mechanisms of neural invasion and the factors that trigger intense immune reactions are not fully understood. Based on recent publications, this review discusses the role of the immune response, interactions between viruses and glial cells, and cytokine mediators in the development of inflammatory diseases in the central nervous system. It also highlights the significant gaps in knowledge regarding these mechanisms.

## 1. Introduction

Accumulating evidence has linked the role of neurotropic viruses in the development of encephalitis and encephalomyelitis, yet the origin of 37% of acute encephalitis cases remains unclear (Solomon et al., 2007). While the currently reported incidence of viral encephalitis is approximately five cases per 100,000 persons, primarily in young and old people (Said and Kang, 2022), it is believed that viral infection can commonly reach the central nervous system (CNS) (Bookstaver et al., 2017). Indeed, numerous viruses, such as Herpesviridae, enterovirus, arbovirus, rhabdovirus, paramyxovirus, and retrovirus, can infect the CNS and lead directly or indirectly to various symptoms. Those manifestations range from meningitis to encephalomyelitis either immediately or delayed from a few days to several years after infection (Bookstaver et al., 2017). Despite the sophisticated blood-brain barrier isolating the CNS from immune cells, neurotropic pathogens have developed strategies to breach these shields and infiltrate the neural parenchyma. Neuroinflammatory cascades triggered by viral infections result in blood vessel injuries associated with rapid and severe-to-fatal harm or brain damage. Fever, headache, motor dysfunctions, neurological and cognitive alterations, or even electroencephalogram (EEG) perturbations are associated with viral encephalitis. The specific symptoms can vary depending on the viral family (Solomon et al., 2012).

Some studies suggest that up to 70% of the confirmed cases of encephalitis could be due to viral infection (Said and Kang, 2022). Numerous flaviviruses, such as West Nile virus (WNV), Usutu virus (USUV), and tick-borne encephalitis virus (TBEV), can reach the neural parenchyma and cause neuronal defects from mild fever to severe neurological disorders, including meningitis, encephalitis or meningoencephalitis (Clé et al., 2020). Human T-Leukemia Virus (HTLV)-1 is responsible for several cases of encephalitis and encephalomyelitis. HTLV-1 prevalence is reported at over 10 million carriers worldwide, although it is underestimated according to the World Health Organization (WHO) (Martin et al., 2018). While the prevalence of Herpesviridae exists in more than 90% of the population, most of the other viruses mentioned so far are emerging or reemerging, sometimes with the ability to spread to more than half of the world population. Altogether, the prevalence of viral infections that commonly infect people and the potential for those viruses to affect the nervous system feature a significant risk for the general population to develop diseases associated with the CNS.

In this review, we report and discuss the most relevant and recent literature on viral-induced neuroinflammatory pathologies. We additionally describe the mechanisms by which these viruses are responsible for either acute or chronic neuro infections, associated or not with detectable inflammation, but leading to fatal late disease. We explain how direct infection and bystander activation seem to be common mechanisms supporting the development of viral-induced neuroinflammation and discuss the different routes of entry into the CNS.

## 2. From infection to CNS reach out

**FIGURE 1:**
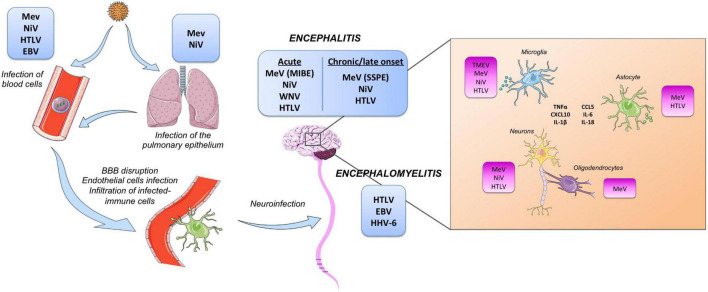
Graphical representation of viral-induced neuroinflammation. **(Left)** Illustration of the route of infection through the nasopharyngeal route or blood fluids into the circulating system and the entry into the central nervous system (CNS) commonly by disruption of the blood-brain barrier (BBB). **(Right)** Illustration with the main viruses infecting the different CNS resident cells and the inflammatory response produced by oligodendrocytes, astrocytes, and microglia to viral infection.

Viruses can use multiple non-exclusive mechanisms to reach the CNS (Figure 1). For example, HTLV-1 transmission occurs via contaminated body fluids. The virus infects the immune cells in the periphery before reaching the neural parenchyma (Rocamonde et al., 2019). Two possible and non-exclusive mechanisms or routes of entry have been proposed. The first proposed mechanism is called the Trojan’s horse strategy using immune cells. Dendritic cells, lymphocytes, and macrophages continuously monitor the CNS aiming to detect agents or pathogens that will endanger neural homeostasis (Ousman and Kubes, 2012). HLTV-1 infects these immune cells and as such, could employ infected-immune cells as a vehicle to infiltrate the CNS and infect neural resident cells. A second proposed mechanism is that HTLV-1 could exploit the presence of HTLV-1 receptors located on the surface of the vasculature to directly infect endothelial cells (Afonso et al., 2008), or indirectly pass through endothelial cells by transcytosis (Martin-Latil et al., 2012), providing a door gate entrance to the CNS. Furthermore, infection of endothelial cells by HTLV-1 would alter the expression of tight junctions thus impairing the functionality of the blood-brain barrier (BBB) and increasing the permeability to lymphocyte passage (Chevalier et al., 2014). These data suggest that both mechanisms can simultaneously infect the CNS.

For other viruses with more commonly observed acute phases, such as Paramyxoviridae or Herpesviridae, multiple exposures and persistence lead to coevolution, allowing the emergence of numerous potential routes of entry into the CNS. However, the relationship between the initial route of entry and the long-term effect of the infection remains largely unknown. In the case of the measles Virus (MeV), the pulmonary epithelium is the first route of entry (Figure 1). Subsequent dissemination through lymphoid tissues is driven by infected lymphocytes. Then, viral transmission to epithelial and endothelial cells is driven by the nectin-4 receptor (Mühlebach et al., 2011; Noyce and Richardson, 2012) and may represent the gateway entry into the CNS by transcytosis (Mekli et al., 2022). Alternatively, the virus can use immune cells and endothelial cells to enter the neural parenchyma. Again, the consequences associated with the route of entry remain poorly documented (Ferren et al., 2019). How MeV reaches and invades the CNS remains unclear, given that there appears to be no high-affinity entry receptor in the CNS.

Nonetheless, infection with MeV can lead to two potentially deadly forms of encephalitis: Measles Inclusion-Body Encephalitis (MIBE) and Sub-acute Sclerosing PanEncephalitis (SSPE). MIBE was reported in an immunocompromised patient after measles-mumps-rubella vaccination. Histological analysis of a brain biopsy revealed the presence of MeV viral particles in neurons, astrocytes, and oligodendrocytes. Indeed, paramyxovirus nucleocapsids were shown to accumulate in inclusion bodies in the cytoplasm of infected cells (Bitnun et al., 1999). In contrast to MIBE, SSPE appears to develop in immunocompetent subjects. Interestingly, viral sequences are not always detected in the brain of patients with MeV-driven acute encephalitis (Ferren et al., 2019).

Natural Nipah virus (NiV) infection starts with a flu-like state, or more rarely, with severe acute respiratory syndrome. NiV targets epithelial, endothelial, or neural cells (Munster et al., 2012; Mathieu and Horvat, 2015). In hamster models, intranasal infection was notably associated with rapid entry and dissemination within the CNS (Munster et al., 2012). In this specific context, the virus directly infected exposed neurons of the olfactory bulb, with anterograde transport being the possible mechanism used by NiV to reach and spread into the ventral cortex. However, the almost systematic generalized vasculitis seems to privilege the endothelial route through a mechanism of transinfection (Mathieu et al., 2011; Matsuura et al., 2015). Furthermore, and in contrast to MeV, NiV shows a low tropism for immune cells. *In vitro* studies have reported the susceptibility of monocyte-derived dendritic cells (MDDC) to NiV infection. Other authors have reported evidence of *in vivo* infection in pigs and hamsters of alveolar macrophages (Wong et al., 2003; Stachowiak and Weingartl, 2012; Baseler et al., 2016). Accordingly, NiV antigens are found in the microvasculature of many organs, but the highest viral load is found in neurons (Wong et al., 2002). NiV-positive neurons were found in the cerebral cortex, frontal lobe, brainstem, and cerebellum, featuring very little necrosis and a poor association with thrombosis. Even if NiV antigens were found in white matter plaques, adjacent glial cells presented low viral load, suggesting neurons and endothelial cells as the main target cells within the CNS. Strikingly, other authors have reported thrombosis and necrosis of neural tissue that was associated with the presence of viral antigens in subjects infected with NiV (Ong et al., 2022). Finally, a study conducted in pigs showed neuroinvasion with NiV after intranasal, oral, and ocular infection. The viral distribution from lung to brain and cerebrospinal fluid, in both sick and healthy animals, pinpointed viral invasion of the CNS using cranial nerves and the trigeminal ganglion in addition to the hematogenous route (Weingartl et al., 2005). However, except for the involvement of a few specific host factors (*e.g.*, RAB11), the way MeV and NiV ribonucleic complexes move within neurons remains poorly documented.

In contrast, Herpesviridae dissemination through axons has been extensively studied and illustrated as an efficient way to invade CNS while being hidden from the immune system. Notably, Alpha-herpesvirus, such as HSV1, can control directional spread in neurons and thus, progress in the neuronal networks by both anterograde and retrograde routes (Enquist et al., 2002; Diefenbach et al., 2008; Ekstrand et al., 2008; Taylor and Enquist, 2015). Rabdoviridae also uses axons and anterograde transport to invade CNS, while the contribution of this entry route for Flaviviridae, such as West Nile Virus (WNV), was suggested but remains less clear (Taylor and Enquist, 2015).

## 3. Immune-based CNS inflammation

Astrocytes and microglia are the main immune effectors within the CNS, displaying a first antigen non-specific response based on the rapid production of pro-inflammatory cytokines. This response is mediated by the recognition of ‘danger’ signals and pathogen-associated molecular patterns (PAMPs) by pattern recognition receptors (PRRs) including toll-like receptors (TLRs) (Bsibsi et al., 2002). Viral genetic material and envelope glycoproteins sensed by TLR can lead to type-I interferon (IFN) secretion and trigger the transcription of interferon-stimulated genes (ISG) involved in the immune response (Hiscott, 2007; Assil et al., 2019). Similar to dendritic cells, glial cells of the CNS express various TLRs that upon viral sensing can trigger IFN-I production (González-Navajas et al., 2012; Kagan and Barton, 2015; Yoneyama et al., 2015). Of note, TLRs play both pathogen-dependent and physiological pathogen-independent roles in glial cells, *e.g.*, homeostasis balance, plasticity, cellular migration, and repair processes (Kielian, 2006; Carpentier et al., 2008). In addition to IFN-I production upon sensing several RNA and DNA viruses, primary cultures of microglia and astrocytes produce cytokine mediators, such as interleukin (IL)-6, tumor necrosis factor (TNF)-α, and IL-1β (Chauhan et al., 2010; Furr and Marriott, 2012; Crill et al., 2015). TNF-α and IFN-γ secretion induced oligodendrocyte apoptosis and inhibited the proliferation of oligodendrocyte progenitor cells (OPCs) (Shi et al., 2015). In contrast, secretion of the neuroprotective IL-6 by astrocytes (Jung et al., 2011; Amantea et al., 2015) enhanced survival and promoted oligodendrocytes differentiation (Pizzi et al., 2004). This exemplified the active participation of astrocytes in both innate and adaptive immune responses and their behavior as effector cells (Dong and Benveniste, 2001), but their functions go far beyond the immune response. They participate in the BBB restoration (del Zoppo, 2009), releasing neurotropic factors (Barreto et al., 2011), and they regulate glutamate uptake and sodium/potassium-ATP activity (Stanimirovic et al., 1997). Thereby, the balance between pro-inflammatory and anti-inflammatory mediators secreted by astrocytes and microglia is critical in demyelination diseases.

In parallel to their ability to secrete cytokines, microglial cells acquire an amoeboid morphology in response to injury or pathogens, a phenotype characteristic of their phagocytic status (Stence et al., 2001). Along with activation, microglia are polarized into a pro-inflammatory (M1) or anti-inflammatory (M2) profile and they act as antigen-presenting cells (Stence et al., 2001; Smith et al., 2012; Perry and Teeling, 2013). Mice infection with Theiler’s murine encephalomyelitis virus (TMEV), showed persistent infection of microglial cells and CD4^+^-T-cells-mediated immune response to myelin epitopes presented by microglial cells, resulting in autoimmune demyelinating disease (Gerhauser et al., 2019). However, the role of microglia in several other viral-induced neuroinflammatory pathologies remains unclear, although they might share similar mechanisms. Interestingly, the presence of activated microglia within demyelinated affected regions has been reported in several non-viral neurological diseases, *e.g.*, amyotrophic lateral sclerosis (ALS) (Sargsyan et al., 2005), multiple sclerosis (MS) (Napoli and Neumann, 2010) or Parkinson’s disease (Long-Smith et al., 2009), suggesting that activation of microglia could be a common mechanism responsible for demyelination in viral and non-viral diseases. Finally, microglial recognition of “eat-me” or “do-not-eat-me” signals presented by neurons can regulate the engulfment of debris or infected cells (Fuhrmann et al., 2010; Neher et al., 2011) and could negatively impact their inflammatory status contributing to sustained inflammation (Neher et al., 2011).

The CNS expression of MHC-I was thought to be restricted to the developing brain or under viral infections (Lampson, 1995; Oliveira et al., 2004). However, MHC-I mRNA expression has been detected subsequently in human dopaminergic neurons from substantia nigra and locus coeruleus norepinephrinergic neurons under homeostatic conditions (Cebrián et al., 2014), supporting a role of antigen-specific cytotoxic responses in CNS damage. Accordingly, antigen-driven MHC-I and CTL-mediated attacks against neurons and astrocytes have been proposed as the etiology of Rasmussen’s encephalitis (Schwab et al., 2009). This might also be the case in HTLV-1 infection, where HTLV-1 Tax-specific CTLs were found in the spinal cord of HAM/TSP patients (Matsuura et al., 2015). HTLV-1-specific CTL recognizes the viral Tax antigen mediated by the Major Histocompatibility Complex I (MHC-I) and induces cytotoxicity of infected cells (Jacobson et al., 1990; Bangham, 2008; Kattan et al., 2009), suggesting a role in bystander damage on CNS resident cells. Recently, microglial cells and neurons have been found positive for HTLV-1 infection (Rocamonde et al., MBio, in press). However, it is still not clear whether microglial positive cells could result from the engulfment of infected cells. Strikingly, the HTLV-1 footprint is absent in the brain of patients with CNS symptoms, suggesting an earlier elimination of infected neurons, probably before symptoms onset. This hypothesis is supported by observations obtained in VSV infection. Indeed, the lack of viral signature in the CNS is still associated with permanent neuronal serotonin reduction due to the immune-mediated elimination of serotonin and norepinephrine neurons selectively infected by VSV (Mohammed et al., 1992; van den Pol, 2009).

## 4. Acute CNS infection

Acute CNS infection is challenging to document, specifically when the viral invasion is rapid due to high neurotropism. Screening of viral genes in the CSF of patients with acute encephalitis can help identify subjacent viral infections not detected in the blood. Several authors have performed PCR analysis on DNA extracted from CSF samples from suspected cases of neurological diseases, detecting the presence of viral genes of HSV-1, HSV-2, HH-3, and VZV in approximately 10% of the samples (Davies, 2005; Guan et al., 2016). The detection of viruses was close to the onset of neurological symptoms. Davies and colleagues elegantly discussed both the advantages and the limitations of the use of CSF samples for the detection of viral infections (Davies, 2005). As previously discussed, viral invasion can utilize various mechanisms that may ultimately lead to acute encephalitis.

In the case of NiV infection in humans, neutrophils, macrophages, lymphocytes, and reactive microglia are the main inflammatory cells found in CNS post-acute encephalitis (Wong et al., 2002). While the virus seems to enter the CNS through a neuronal anterograde transport (see previous section), the large representation of the BBB alteration strongly suggests the hematogenous route is likely responsible for the acute CNS manifestation of NiV infection. Indeed, despite multi-organic infection, NiV seems to target the vascular system and creates profound damage, increasing the size of blood vessels (Wong et al., 2002). Systematic fever is one of the hallmarks of NiV infection and may be accompanied by strong migraines. Dissemination of the NiV from the immune system is performed by *cis*- and *trans-i*nfection leading to systemic vasculitis. With an average of 9.5 days post-infection, serious brain damage can be caused by NiV endothelial infection ranging from loss of consciousness up to death in 40-100% of cases (Lee, 2007). The neural invasion itself can lead directly and indirectly to irreversible damage (Ong et al., 2022). Infection of endothelial cells and CNS invasion are associated with TNF-α, IL-1β, and CXCL10 secretion, which correlate with the recruitment of immune cells in the brain parenchyma (Mathieu et al., 2012; Escaffre et al., 2013).

Conversely, MeV mainly targets activated immune cells during the acute phase of the disease, leading to diverse responses in immunocompetent patients. Among them, acute post-exposure encephalitis is generally observed within a few days post-infection. Uncontrolled inflammation triggered by infection leads to an incorrect immune response often compared to autoimmune-like encephalitis and, in most cases, unrelated to the viral presence in the CNS parenchyma (Ferren et al., 2019). In contrast, in immune-compromised patients, viruses can enter and spread into the CNS, mainly but not systematically because of viral mutations affecting the matrix protein and the fusion machinery (Mathieu et al., 2021). In this case, mutations destabilize the fusion complexes driving the entry, leading to infection of any cells independently of known high-affinity receptors, those binding by MeV envelopes trigger the exposure of viral fusion peptide allowing viral entry of wild-type MeV. While neurons seem to be the privileged cell network for dissemination, MeV seems able to enter and replicate in the four main cell types from the CNS (*i.e.*, neurons, astrocytes, oligodendrocytes, and microglial cells) (Figure 1). Infection of CNS resident cells leads to antiviral responses, notably type-I IFN and astrogliosis, which combined with the natural cytopathic effect of the virus, leads to profound CNS damage (Welsch et al., 2019; Mathieu et al., 2021).

WNV commonly uses retrograde transport and thus infects neurons in addition to endothelial cells, similar to Henipavirus. Microglial cells surrounding infected neurons also change their morphology to an activation profile. This activation profile is associated with an upregulation of pro-inflammatory cytokines, such as CXCL10, IL-6, CCL5, and TNF-α, involved notably in the recruitment of T-cells (Jin and Yamashita, 2016). Studies recently reported that the removal of microglial cells in the early stages of WNV infection exacerbates infection and may be associated with the antigen presentation role of microglial (Wheeler et al., 2018). Indeed, microglia first limit viral growth and reduce mortality as observed experimentally in mice (Stonedahl et al., 2020). However, in the later phases of the infection, T-cells recruited in the brain parenchyma activate microglial cells, which leads to neuronal alteration, such as synapse disruption, and causes long-term neurological deficits in surviving patients (Klee et al., 2004). One theory tends to suggest that microglial cells directly recognize WNV through TLR3, leading to initiation of antiviral response with cytokine secretion and recruitment of CD8 + T-cells essential for the elimination of the infection, yet causes collateral damage to the tissue (Garber et al., 2019). These CD8 + T-cells also seem to differentiate in memory T-cells when they enter the brain and promote microglial cell-mediated synaptic elimination during viral infection (Chen et al., 2019). The persistence of neuroinflammation caused by the presence of T-cells in the CNS over a period of years may also have negative effects in case of other viral infections that reach the neural tissue.

Acute encephalitis is a rare event in HTLV-1 infection, for which the development of HTLV-1-associated myelopathy/tropical spastic paresis (HAM/TSP) is more commonly reported. This is associated with sustained HTLV-1-driven neuroinflammation that appears years after asymptomatic infection. However, several cases of HTLV-1-related acute encephalitis have been reported over the past years suggesting a different mechanism of infection/response (Araga et al., 1989; Smith et al., 1992; Tachi et al., 1992; Tateyama et al., 1999; Puccioni-Sohler et al., 2007). Unlike HAM/TSP, here, HTLV-1-driven encephalitis develops within months to a few years after the initial infection. Crawshaw reviewed three cases of HTLV-1 encephalitis in women from 35 to 52 years old with rapid evolution that led to a fatal outcome. Two of the three patients showed no previous white matter lesions at the onset of symptoms. A few months later, white matter lesions were observed and both hypothermia and hyperthermia were associated with hypothalamic damage. In some of these patients, there were waves of neurological episodes with white matter lesions, neurological symptoms, and motor dysfunctions – including disorientation, fever, aphasia, loss of consciousness, paraparesis, and tremors – that were relieved temporarily with anti-inflammatory treatments (Crawshaw et al., 2018). However, in some cases treatment with steroids was unsuccessful in stopping disease progression, resulting in a fatal outcome. Notably, higher levels of HTLV-1 proviral load were found in cerebrospinal fluid (CSF) compared to peripheral blood mononuclear cells (PBMCs), which appeared to be associated with encephalitis episodes (Crawshaw et al., 2018).

## 5. Long-term neuroinflammation

### 5.1. Chronic/Relapse

In approximately 5% of HTLV-1-infected subjects, unsolved sustained neuroinflammation led to demyelination of the spinal cord and the paralysis of the lower limbs (Gessain et al., 1985; Izumo et al., 1992; Nagai and Osame, 2003). HTLV-1 has also been linked with other neuropathies, such as acute disseminated encephalomyelitis, meningitis, myopathies, or peripheral neuropathies, whose development is still not well understood (Khan et al., 2017). Strikingly, magnetic resonance imaging (MRI) revealed lesions in other areas of the CNS, such as cortical white matter regions, even before symptomatic manifestations (Kitajima et al., 2002; Morgan et al., 2007).

Yet, the etiology of neuroinflammation reported in HTLV-1 neuro infection remains elusive. Infiltrated cytotoxic T-cell lymphocytes (CTL) are frequently found in spinal cord lesions of HAM/TSP patients (Matsuura et al., 2015). Infiltrated CTL expressed proinflammatory cytokines including IFN-γ, TNF-α, and IL-1β. The role of IFN-γ in the neuroinflammatory loop seems to be central. IFN-γ release stimulates the production of CXCL10 by astrocytes (Ando et al., 2013), which in turn induces the migration of CXCR3 + inflammatory cells (Rafatpanah et al., 2017; Yamano and Coler-Reilly, 2017). However, what triggers CTL infiltration in the first place is not yet well understood.

Several authors have attempted to identify predictive biomarkers for HAM/TSP development. The landscape of cytokines and chemokines analyzed by multiplex screening performed in serum and CSF of HTLV-1-infected individuals suggested no correlation with the speed of HAM/TSP progression. However, the concomitant increase of IL-18 levels in CSF indicates the involvement of inflammasome in the development of HAM/TSP. Simultaneously, low levels of TGF-β1 detected in the very same samples suggest an impaired immunosuppressive response to control neuroinflammation (Freitas et al., 2022). Immunoprofiling of HAM/TSP blood samples showed increased responsiveness of innate cell subsets producing high levels of pro-inflammatory cytokines (Rocamonde et al., 2021). High concentrations of CXCL10, neopterin, and neurofilament light (Nf-L) were found in the CSF of patients with severe diseases that rendered them wheelchair-dependent. CSF levels remained elevated in follow-up analyses of plasma and CSF samples (mean follow-up 5.2 years), correlating with neuronal damage and sustained neuroinflammation (Rosadas et al., 2020). Concomitantly, CSF levels of neopterin and CXCL10 were demonstrated to predict the speed of HAM/TSP progression better than Nf-L (Souza et al., 2021).

Similarly, higher serum concentrations of pro-inflammatory cytokines (TNF-α, IFN-γ, IL-1β, IL-6, and CCL-5) were detected in HHV-6 seropositive multiple sclerosis (MS) patients compared to HHV-6-seronegative subjects and correlated with the mean values of the expanded disability status scale (EDSS) (Keyvani et al., 2020). These data support the hypothesis of early HHV-6 infection playing a role in MS pathogenesis or, at least, in the severe MS forms by maintaining a sustained inflammatory status.

Early infection of children by MeV results in a high probability of developing SSPE within the two decades following primary infection. SSPE is generally associated with chronic neuroinflammation leading to neuronal loss and demyelination. For SSPE diagnosis the presence of anti-MeV IgG antibodies in the CSF is essential. They could assign the presence of residual viral particles that would remain latent in the CNS and get reactivated years later. Accordingly, infiltrated mononucleated immune cells are commonly observed together with glial reaction and infection of microglial cells (Anlar et al., 2003; Garg, 2008; Welsch et al., 2019). Monocytes and CD4 + T-lymphocytes are observed in the perivascular areas and CD8 + T-lymphocytes were more frequently observed in the parenchyma.

Individuals who survive Nipah virus infection develop a persistent form of severe fatigue, along with neurological and motor dysfunctions (Sejvar, 2007). White matter regions seem to be mainly affected (Ng et al., 2004). In primates experimentally infected with NiV, the persistence of the infection also occurred. NiV antigens were mainly detected in neurons and microglia of the brainstem, cerebellum, and cerebral cortex (Liu et al., 2019). Thus, the persistence or appearance of abnormalities, such as cognitive dysfunction or intellectual disability, would be the direct consequence of acute encephalitis. Indeed, patients developing this type of symptoms subsequently present cerebellar atrophy, brainstem lesions as well as abnormal neural transmission at the cortical level. In 9% of infected subjects, the neurological symptoms appear as a consequence of relapsed encephalitis within 48 months of primary infection (Tan and Wong, 2003). In Malaysia, nearly 7.5% of patients who survived acute encephalitis relapsed within a year of infection. Even though none of these individuals had been re-exposed to the infected pigs they still had anti-Nipah IgG in the serum and in some cases IgM (Tan et al., 2002).

### 5.2. Persistency/Late onset link to asymptomatic infection

Viral-driven neuro infection is not always presented together with visible symptomatology. NiV, HTLV-1, as well as potentially HBV and EBV, can also lead to asymptomatic cases. In Malaysia, a nurse exposed only to infected patients seroconverted to henipavirus and also showed brain lesions visible on an MRI scan without any apparent signs of infection (Ali et al., 2001). Another characteristic of NiV is its ability to cause late encephalitis. Indeed, in nearly 3.5% of cases, patients who presented with an acute non-encephalitogenic infection or who were asymptomatic, declared encephalitis more than 8 months after the initial infection. These data suggest that NiV can persist in a latent form in the CNS for several months and even up to 11 years, before reactivating, thus causing the relapse or late development of acute encephalitis, which can be fatal as well (Abdullah and Tan, 2014). Some studies even suggested that approximately 16% of asymptomatic, seropositive individuals monitored by MRI scans exhibited lesions in their brains (Ong et al., 2022). This is slightly less than the number of patients with acute NiV encephalitis. These data demonstrate the importance of monitoring exposed people, even several months after contact, whether hypothetical or confirmed. This reactivation could be due to environmental factors or to transient immunosuppression of patients.

Research conducted on Grivets primates has also shown that late onset of encephalitis is possible. In these studies, persistent NiV infection was only detected in the brain, specifically in neurons and microglial cells of the brainstem, cerebral cortex, and cerebellum. The main pathway for initial entry was via the endothelia during the acute phase of the infection, but this route was no longer observed in the late phase (Liu et al., 2019). Encephalitis among survivors was characterized by lymphohistiocytic inflammation associated with necrosis, rarefaction, spongiosis, and microglial cell activation. Notably, proliferating Ki67^+^ cells, CD68^+^ resident microglial cells together with a few IgG and CD20^+^ B-cells, highlight the increased permeability or permissiveness of the barriers found in the parenchyma.

In contrast, around 70% of patients exposed to NiV develop IgM and IgG antibodies directed against the virus and 30% of the patients showed the presence of antibodies in CSF (Goh et al., 2000). This suggests an immune response is mounted against the virus and opens prospects for vaccination and protection. Yet even if high levels of antibodies are detectable this might not be protective. For example, a 12-year-old child, exposed to NiV in December 1998, did not develop encephalitis until 4 months later. The initial IgG titer when admitted to the hospital was high and while doctors expected this titer to drop, it continued to rise for the next 2 weeks. The profile of IgG1 and IgG3 in this patient was found to be very similar to that observed in particular cases of SSPE (Wong et al., 2001).

SSPE is generally observed either in young patients or in young adults (20-35 years old) (Garg, 2008) when the primary infection occurred during the first two years of life when the immune system is still immature (Griffin et al., 1987). The presence of residual maternal antibodies has been suggested to be a potential factor leading to persistency. However, it remains unclear where exactly and how MeV could hide up to 23 years post-infection in the CNS without any visible symptoms. To date, the potential link between the measles paradox in specific patients and late onsets such as SSPE has never been explored (Poland and Jacobson, 1994). In a large part of the viral sequences retrieved from the brain of patients who died from SSPE, mutations and hypermutations were almost systematically founded in the MeV matrix protein as well as in the fusion complex leading to hyperfusogenic phenotype (Angius et al., 2019; Ferren et al., 2019). Currently, the aggressiveness of the hyperfusogenicity seems incompatible with the ability to remain dormant within the CNS without any symptoms, suggesting that the virus may have evolved in the tissue under specific selective pressures. For alpha-herpesvirus HSV1, spread from peripheral axons to epithelial cells is a major way of inter-host dissemination after latency (Taylor and Enquist, 2015), one could ask whether a similar system could exist for paramyxovirus. In this case, the virus would remain hidden from the immune system and out of the CNS for years and would only invade the CNS through axonal transport just before the declaration of the late onset (for Nipah virus) or SSPE (for MeV). In addition, the transport may be associated with cytokines release triggering inflammation and immune responses against the CNS, thus fostering the aggression of neural cells.

SSPE is mainly associated with perivascular CD4 + T-cells and B-cells infiltrates (Garg, 2008). IgGs are systematically present in the CSF. Necrosis and astrogliosis are observed in the parenchyma (Garg, 2008; Welsch et al., 2019). Antigens were found in microglial cells (Mesquita et al., 1998), however, it remains unclear whether these cells were infected or if they have phagocyted infected cells. Both reactive astrocytes and CD68^+^ hematogenous monocytes and perivascular microglia are often observed close to antigens expressed on neurons in humans, *in vivo* as well as *ex vivo* with potential consequences on inflammation and neurodegeneration (Mesquita et al., 1998).

In contrast to the viruses previously mentioned, HTLV-1 has a longer onset period in the development of human T-cell leukemia/lymphoma (HAM/TSP) with an average of 20 years between infection and the onset of first symptoms. However, positron-emission tomography (PET), which assesses microglial activation, has demonstrated significant neuroinflammation in the brain of asymptomatic HTLV-1 carriers although to a lesser degree than that of HAM/TSP patients (Dimber et al., 2016). Furthermore, MRI has revealed lesions in several CNS areas, such as cortical white matter regions, even before symptomatic manifestations with no evident symptomatology (Khan et al., 2017). This suggests early viral invasion and persistence in the brain of asymptomatic carriers with chronic but not-yet pathogenic neuro-inflammation.

Both HAM/TSP and MS share common pathophysiological features, with neurodegeneration as the consequence of sustained neuroinflammation. Indeed, only serological tests for HTLV-1 can make a differential diagnosis of HAM/TSP in contrast to MS. While HAM/TSP sustained neuroinflammation is triggered by HTLV-1, so far, no etiological agents have been directly linked with MS development. However, some common herpesviruses have been proposed as potential candidates. Although all of them are highly prevalent in the world population, it has been proposed that MS susceptibility is developed during infancy. In addition, the HHV-6 virus can easily evade the immune system and establish a long latency period and its reactivation has been linked with MS clinical relapses (Virtanen and Jacobson, 2012). HHV-6 can infect a wide range of cells including different CNS cell types. HHV-6 reactivation has been reported in several cases, notably in immunosuppressed hosts, and it has been related to cases of severe chronic neurological diseases, such as MS and epilepsy (Challoner et al., 1995; Donati et al., 2003).

Viral triggering of MS has also been recently illustrated by the detection of Epstein Barr Virus (EBV) in the brain (white matter and meninges) of 90% of MS brain samples using PCR and EBER-*in situ* hybridization (EBER-ISH) (Hassani et al., 2018). None of the samples were positive by PCR for other common herpesviruses (HSV-1, CMV, and HHV-6). Although in most cases the EBV viral load was low to moderate, in 18% of multiple sclerosis (MS) cases the virus was widespread within the central nervous system (CNS), and expression of the EBV latent protein EBNA1, and to a lesser extent the early lytic protein BZLF1, were detected. EBV infection was also reported in astrocytes and microglial cells in MS patients (Hassani et al., 2018), suggesting that a common mechanism of viral clearance by the CNS could be at the onset of neuroinflammation, as was described above for other viruses.

## 6. Conclusion

Many of the viral-driven neuro infections described above can lead to various forms of persistence, which can result in insidious or late, but still acute, clinical manifestations with irreversible consequences that can be fatal. Whether these viruses remain or not hidden for months to years in the CNS remains extremely difficult to confirm due to the lack of tools allowing the real-time visualization of the virus in vital organs such as the brain. It is not understood well how viruses such as Paramyxovirus NiV or MeV, known for their high cellular cytopathic effect mainly related to the instability of their fusion glycoproteins, can remain dormant for years without causing cell fusion and syncytia, yet a general feature of infection. Moreover, how suddenly those viruses re-emerged with hyperfusogenic phenotypes is not understood. For others, such as HTLV-1, EBV, or HHV-6, the drivers of entering the CNS and how infected cells leave blood circulation remain poorly understood.

However, all these viruses, by their presence or not in the CNS, seem able to induce the secretion of cytokines, either by endothelial cells or by immune cells, leading to the reactivity of astrocytes and microglial cells typically associated with viral encephalitis or encephalomyelitis. Unfortunately, it remains very difficult to investigate the interaction between viruses and microglial cells in the CNS context during the early phases of neuroinvasion and how these interactions could lead to acute or persistent infection. Thereby, the generation of accurate *in vitro* systems is of utmost importance to understand such events. As such, organotypic brain cultures are the only tridimensional systems, which include endothelial cells as well as the four neural cell types in their native organization, allowing visualization of the early phases of the infection (Welsch et al., 2019; Ferren et al., 2021). However, after a week of culture, the slicing procedure leads to the activation of the astrocytes and microglial cells and tridimensional reorganizations making long terms investigations of such interactions difficult to perform (Welsch et al., 2019). Alternatively, three-dimensional (3D) neural ‘mini-brains’ or human brain organoids (hBORGs) recapitulate many features of the neural cell diversity and the complex organization of the human brain and represent a step forward to 2D cultures. hBORGs exemplify the ambition to recreate more accurately physiological conditions, dynamic of circulating molecules, extracellular matrix, and spatial arrangements between neighboring cells. However, hBORGs consist for the moment of an assembly of differentiated cells from single tissues, thus lacking microglial cells or endothelial cells, or both. Thus, while they present the evident advantage of working with human models for evaluations of cell-to-cell dissemination in neuronal networks or astrocyte activation (Mathieu et al., 2021), their low level of complexity still does not allow addressing the mechanisms involving all cellular actors of the CNS inflammation. Accordingly, most of the information about late stages or long-term interaction between virus and CNS remains MRI data (Ong et al., 2022) and postmortem histology analysis, often mainly focused on areas showing lesions with limited understanding of the pathogenesis. Here, we discussed that virus infection could lead to an inadequate destructive response far from the primary site of infection.

Therefore, future research should focus primarily on the mapping of viral trafficking and tropism during the progression of the infection and before symptoms onset and, secondly, on identifying host factors shared among individuals who develop encephalitis or encephalomyelitis. In this context, microglial cells’ activation and response against the infection may be a key crucial factor in the development of acute or sustained neuroinflammation.

## Author contributions

BR, CM, and HD contributed to the conception, writing initial draft and edition, and figure drawing. UH contributed to the writing and figure edition. All authors listed have made a substantial, direct, and intellectual contribution to the work, and approved it for publication.
